# Saudi students’ perspective towards language education policy of using the first language in teaching the second language

**DOI:** 10.1186/s40862-022-00146-5

**Published:** 2022-07-01

**Authors:** Thamir Issa Alomaim, Yaser Mohammed Altameemi

**Affiliations:** English Language Department, College of Arts, University of Hail, Hail, Saudi Arabia

**Keywords:** Language education policy, First language, Second language, Preparatory year program, Students’ perspective

## Abstract

This is a mixed methodology study that explores language education policy (LEP) of using the first language (L1) in teaching the second language (L2). The literature points out that students’ perspective towards LEP of the preparatory year program (PYP) in Saudi universities has not been investigated before. This study engages with understanding students’ awareness about LEP of the PYP towards L1 and students perspectives towards using L1 in teaching L2. Students from all the tracks of the PYP, namely: medical, science and humanities tracks participated in this study. This study used interviews and questionnaires as tools to collect data. Data collection of this study was classified into two phases. The first phase was in 2015 and the second phase was in 2020. Data analysis of this study reveals that the majority of students in the first and second phases were not familiar with the LEP of the PYP towards L1. Data analysis points out that students’ perspective towards using L1 in teaching L2 did not correlate between the first and second phases of this study. Most of the participants in the first phase did not support using L1 in teaching L2. On the other hand, the majority of the participants in the second phase supported using L1 in teaching L2. Policy-makers in the PYP are encouraged, based on the results of this study, to adopt an “English-mainly” policy that considers students’ perspective in the LEP of the PYP and treats students as bilingual speakers of both the Arabic language and the English language.

## Introduction

The role of the English language in the education sector is increasing globally. This is a result of its significant influence in various fields that include science, economy, entertainment and so forth. This expanding influence led countries such as Saudi Arabia, the context of this study, to use the English language as a main medium of instruction in its undergraduate education. The main medium of instruction in Saudi public schools is the Arabic language while the main medium of instruction in the universities is the English language. Therefore, the vast majority of Saudi universities implemented a preparatory year program (PYP) that is intended to prepare students for their undergraduate education and bridge the gap between public education and undergraduate education. The PYP includes an intensive course in English as a foreign language (EFL) in addition to several other modules that use the English language as a medium of instruction. Passing the PYP is a mandatory prerequisite for students to be permitted to enroll in undergraduate education. Due to COVID-19 pandemic, the education sector in Saudi Arabia, including the PYP in Saudi universities, has shifted to distance learning to control the spread of the virus.

The literature reveals that students’ perspective on language education policy (LEP) towards using the first language (L1) during teaching the second language (L2) has not been studied before in the context of teaching EFL in Saudi universities (Almoaily & Alnasser, 2019; Almulhim, 2014; Alomaim, 2019; Alrabai, 2019; Alshammari, 2011; Alsuhaibani, 2015; Faruk, 2014; Habbash, 2011; Pavan, 2016). A great deal of research has been conducted in Saudi Arabia on LEP with a particular focus on the role of EFL in the field of education. For example, Almoaily and Alnasser (2019) suggest implementing policies in the English language departments of the Saudi universities that maximize the opportunities for English language practices and include non-Arabic Speakers. Alomaim (2019) conducted a case study to investigate the LEP of a PYP towards using L1 in teaching L2 in a Saudi context. He discovered that the LEP of the PYP bans L1, but the EFL teachers were not aware of this ban. He revealed that the EFL teachers did not support or implement this ban on L1. This study aims to explore students of the PYP perspective in regards to the LEP towards using L1 in teaching L2. The current study is a case study that will expand (Alomaim, 2019) study through investigating students of the PYP’s awareness about the LEP of the PYP towards using L1 in teaching L2. In addition, this study will also analyze students’ perspective on using L1 in teaching L2. That is, it will detect whether students support using L1 in teaching L2 or not to evaluate students’ awareness about the LEP.

## Literature review

Language policy and language planning are sometimes used as synonyms (Cooper, 1989; Mesthrie et al., 2009). However, they are not exactly the same. Haugen (1959) was the first to use the term language planning. Several definitions of language planning have been proposed (e.g., Cooper, 1989; Haugen, 1959). According to Cooper (1989, p. 45), language planning is “deliberate efforts to influence the behaviour of others with respect to the acquisition, structure, or functional allocation of their language codes.” Cooper’s definition of language planning indicates influencing linguistic behavior without changing it or solving problems (García, 2015). Language planning has been classified into three types: status planning, corpus planning and acquisition planning. The German sociolinguist Kloss (1969 cited in García, 2015, p. 354) introduced the first two types. Status planning includes the process of selecting a language or a variety of it for use whereas corpus planning refers to the process of codifying the selected language or variety. The third type, namely acquisition planning, was created by Cooper (1989); Ferguson (2006) explains that acquisition planning focuses on increasing the numbers of users/speakers of any given language.

Beliefs, practices, and regulations that impact how people use language are examples of multiple forces that may influence behavior toward language (García, 2015). Therefore, García (2015) points out that there were calls by some scholars to recognize these forces through renaming the field of language planning as language policy. Baldauf (2005) defines language policies as bodies of ideas, laws, regulations, rules, and practices that are intended to achieve some planned language change. Language policies exist even if they have not been explicitly mentioned or established by authorities (Bassiouney, 2009; Spolsky, 2004).

Education is considered by language planning as the main field of executing language policy (Hoffmann, 1991; Lo Bianco, 2010). Therefore, LEP should be considered as a branch of language policy/ language planning (Paulston & Heidemann, 2006). Language planners tend to use the educational system to introduce a second or a foreign language (Cooper, 1989). Thus, acquisition planning is a crucial element in all the stages of the educational process starting from the ministry of education to teachers in their classrooms (Cooper, 1989). This led to renaming acquisition planning as language-in-education planning by Kaplan and Baldauf (1997) and LEP by Shohamy (2006). Shohamy (2006, p. 76) states that LEP is “a mechanism used to create de facto language practices in educational institutions, especially in centralised educational systems.” Hence, LEP acts as a tool to promote political, ideological, social and economic agendas (Shohamy, 2006). The main focus of this study is the LEP of the PYP. Studying LEP with a particular focus on the perspective of students would assist policy-makers in improving their policies through considering the role of students.

LEP in countries that adopt centralized educational systems are constituted by central authorities to assist in accomplishing national language policy agendas (Shohamy, 2006). These authorities are represented in Saudi Arabia by the King and the Council of Ministers on the national level whereas the Dean of the PYP, the Director of the ELC and the PYP board represent these authorities on the PYP level.

Forming language policies with regards to the use of L1 and L2 in schools and universities are part of the LEP (Shohamy, 2006). Nonetheless, policy-makers tend to perceive using L1 in teaching L2 in the context of EFL as a form of deficit behavior (Raschka et al., 2009). This perception comes from the wide advocacy of the significance of implementing monolingual language instruction (Raschka et al., 2009).

Although some countries such as Japan and South Korea encounter a contradiction between their official languages and their LEP, their LEP discourages using L1 inside the L2 classes (e.g., Glasgow, 2014; Kang, 2008; McMillan & Rivers, 2011). Turkey adopts in its constitution the Turkish language as its official language. However, the Turkish Council of Higher Education (2014) clarifies that several Turkish universities use the English language as the medium of instruction for around one-third of their modules.

Saudi Arabia in its Basic Law of Governance (constitution) prescribes the Arabic language as its official language. Oman, Abu Dhabi, the capital emirate of the United Arab Emirates, and Qatar prescribe in their constitutions that the Arabic language is their official language. However, in the latter three examples, there are increasing encouragement to improve students’ proficiency in the English language (e.g., Al-Bakri, 2013; Gallagher, 2011; the Qatari Education and Training Sector Strategy 2011–2016).

According to article 24 of Saudi Ministry of Education (1995), “principally, the Arabic language is the language of education in all subjects and all stages unless there is a necessity of teaching with another language.” Article 24 does not name the other language that may be needed in teaching. Nonetheless, only the English language is the additional language that is taught in Saudi public schools. In addition to this, article 11 of the Saudi Council of Higher Education (2007) states that “Arabic is the language of education in universities and it is permitted when appropriate to teach with another language by a decision of the concerned university.” However, the vast majority of Saudi universities have introduced the PYP to provide students with intensive courses in EFL besides other modules that their medium of instruction is the English language. Passing the PYP is a mandatory requirement for students to be allowed to progress to their undergraduate majors. This is because most of the undergraduate majors in the Saudi universities, particularly majors under the umbrella of science and medicine, use the English language as their medium of instruction.

According to Spolsky (2004, p. 9), language practice is “the sum of the sound, word and grammatical choices that an individual speaker makes, sometimes consciously and sometimes less consciously, that makes up the conventional unmarked pattern of a variety of a language”. Language practice is an element that directly relates to language policy (Bassiouney, 2009). Policies are mirrored by classroom practices (Paulston & Heidemann, 2006). On the other hand, the influence of language policy, whether it is formally written or not, on language practice cannot be guaranteed or be consistent (Spolsky, 2004). Language policies are useless without the positive involvement of humans who play a significant role in implementing these policies (Hornberger & Johnson, 2007). It cannot be assured that language policy would be implemented if it contradicts with language practice (Bassiouney, 2009). For example, teachers and learners practice the language (either L1 or L2) with their focus on delivering the required knowledge of the course to overcome the limitations of the language policy (Creese, 2005 cited in Asker & Martin-Jones, 2013, p. 345).

Literature shows emerging calls for LEPs to reconsider the beneficial use of L1 in teaching L2. This is because allowing L2 only by the policy is not practical (Raschka et al., 2009). These calls propose that the policy should consider the possible advantages of L1 (Garton, 2014; Raschka et al., 2009). Allowing L1 may improve students’ learning opportunities (Garton, 2014). Hence, an “English-mainly” policy should be implemented rather than an English-only policy (McMillan & Rivers, 2011). Nonetheless, permitting the use of L1 should not lead to overuse of it that both teachers and learners would rely on L1 (Turnbull & Dailey-O’Cain, 2009).

Code-switching between L1 and L2 bridges the gaps that may occur in the discourse (Moore, 2002). Using code-switching between L1 and L2 assists students in fixing any breaks in the communication that may result from a lack of understanding their teacher. Nonetheless, code-switching has to be controlled by both teachers and students (Cipriani, 2001; McMillan & Rivers, 2011).

Translanguaging has been introduced to move the discussion of the use of L1 by bilinguals beyond calling it code-switching. Translanguaging was first introduced by Cen Williams (1994, cited in Hornberger & Link, 2012, p. 268). According to García (2008, p. 58), translanguaging is “multiple discursive practices in which bilinguals engage in order to make sense of their bilingual worlds.” Translanguaging incorporates code-switching, but it goes beyond it (García, 2008). Alqahtani (2022) found out that using translanguaging in the EFL classes is strongly supported by the Saudi students even though they were worried that it may not assist them in reaching the desired proficiency. However, as Alomaim (2019) points out, the students in the context of this study have low proficiency in L2. Therefore, code-switching will be used in this study instead of translanguaging.

Calls to reconsider learners of L2 as bilinguals instead of encouraging them to disregard L1 during learning L2 has expanded. The global spread of the English language negatively influenced seeking the native-speaker model among many learners of it (Hall & Cook, 2012). Therefore, abandoning the linguistic resources of the learners obstructs their possibilities of multilingual development (Hornberger & Link, 2012). Although learners of L2 will not become monolingual speakers of L2, it is possible for them to become bilingual speakers of the two languages (Liebscher and Dailey-O’Cain, 2005). Poulisse and Bongaerts (1994) argue that bilinguals are able to separate the two languages. However, storing languages in separate compartments in the mind is not a successful strategy because these compartments are connected in several ways (Cook, 2001). Thus, the mind does not store languages in separate compartments. Cook (2012) coined the term multicompetence and defined it as “the knowledge of more than one language in the same mind or the same community”. Multicompetence involves the entire mind of the speaker (Cook, 2013). Multicompetence classifies people into multilingual and monolingual people instead of considering multilinguals as deficient monolinguals (Cook, 2013).

Most of the students in the context of this study speak Arabic as their L1. Thus, this study will consider the role that the Arabic language may play in teaching and learning the English language. There are mainly two types of education in terms of the medium of instruction: monolingual teaching and bilingual teaching. Monolingual teaching refers to the use of the target language alone while bilingual teaching uses two languages that include the target language and a language that students are familiar with (Hall & Cook, 2012).

L1 is a significant tool that facilitates students’ learning of L2 (Butzkamm, 2003). L1 may be used in improving the relationship between teachers and their students (Franklin, 1990; Harbord, 1992; Qian et al., 2009; Tien, 2009). For instance, L1 enhances interactions inside the classroom (Eldridge, 1996; Greggio & Gil, 2007; Harbord, 1992; McMillan & Rivers, 2011; Rezvani & Rasekh, 2011). L1 assists teachers in improving their students’ comprehension (Edstrom, 2006; Kim & Elder, 2005; Liu et al., 2004; Uys & Dulm, 2011). Besides this, it is possible for students to use L1 in translating their teacher’s instruction to their colleagues. This ability indicates a development in students’ proficiency in L2 (Yletyinen, 2004).

On the contrary, using L1 in teaching L2 has encountered heavy criticism. For instance, Ellis (1984) argues that it would deprive students from significant input in L2. For example, some students tend to use L1 in matters that are not relevant to the lesson (Jakobsson & Rydén, 2010). Krashen (1982) suggests that errors that are caused by the use of L1 would restrict production of formally-correct sentences in L2. In addition, schools and teachers tended to consider using L1 in teaching L2 as a negative trait because it may negatively influence one or both of the languages (Hughes et al., 2006). Teachers and students’ use of L1, is usually accompanied by a sense of guilt (Simon, 2001). Teachers and researchers usually claim that students use of L1 during learning L2 is a sign of failure because it would reduce students’ motivation to learn the language and it would not prepare them for face-to-face interaction (Eldridge, 1996; Franklin, 1990).

Nation (2003) states three main reasons for students’ use of L1 instead of L2. L1 is the default language among people who share a common language, L1 improves the efficiency of communication, and L1 is preferred by students who suffer from low-confidence of using L2 in front of others.

Alomaim (2019) asserts that the proficiency level of the students and the difficulty of the discussed topic are major factors that influence the amount of using L1 in the L2 classroom. For example, literature shows that students who have low proficiency in L2 tend to use L1 (Baoueb & Toumi, 2012; Ferguson, 2009; Franklin, 1990; Jakobsson & Rydén, 2010; Liu et al., 2004; Poulisse & Bongaerts, 1994; Simon, 2001; Then & Ting, 2011). Hence, L2-only policy is not practical with students who have low proficiency in L2 (Tien, 2009). However, Alomaim (2019) points out that every language classroom should be considered as an isolated situation which means that deciding an ideal amount of L1 for all language classrooms cannot be achieved.

On the other hand, permitting the use of L1 should not lead to extensive use of it. Researchers indicate that the classroom may act as the mere source of learning L2 for the majority of students (Littlewood & Yu, 2011; Polio & Duff, 1994; Turnbull & Arnett, 2002). Therefore, maximal use of L2 should be implemented inside the classroom (Copland & Neokleous, 2011; Edstrom, 2006; Nation, 2003; Turnbull & Arnett, 2002).

The literature indicates that LEP and the role of L1 in it is a significant issue particularly in the context of teaching L2 (Garton, 2014; McMillan & Rivers, 2011; Raschka et al., 2009). However, the literature reveals that the perspective of students in the LEP has not taken sufficient consideration whether L1 is permitted in teaching L2 or not. Thus, this study aims to analyze students’ perspective towards the LEP in terms of the role of L1 in teaching L2 and whether students agree with using L1 in teaching L2 or not. This is a case study that will study students of a PYP in a Saudi university to engage with answering two significant questions:

1. What is the extent of students’ familiarity with the LEP of the PYP towards using L1 in teaching L2?
2. What is students of the PYP’s attitude towards using L1 in teaching L2?

## Methodology

Alomaim (2019) conducted a study to investigate LEP of the PYP towards L1 from the perspective of the policy-makers and teachers of L2. This study focuses on students’ perspectives towards the LEP of the PYP towards using L1 in teaching L2. This study incorporates a mixed methodology. This study was conducted in the PYP of the same university in Saudi Arabia and its data have been collected during two phases. It was conducted within two phases to detect any changes in the status of LEP in the studied PYP and whether the policy-makers have considered the role of the students in the LEP of the PYP or not. The first phase of this study that was conducted between January and April 2015 and it used interviews. The second phase of data collection included online surveys that was distributed in November 2020. This study had a precondition that all its participants had to be students of the PYP during data collection. Thus, all the participants of this study were students of the PYP in the investigated university.

During the first phase, semi-structured interviews were conducted with seven different students of the PYP. Semi-structured interviews facilitates asking the interviewees particular questions and have the ability of paraphrasing them whenever it is necessary. The interviewed participants were mainly from the science and medical tracks. Students from the male section of the PYP only were asked to participate in the interviews because it was not feasible to include the female section for cultural and administrative boundaries. The interviews were conducted according to the convenience of the students and their class schedule. Some of the participants were interviewed individually and the others were interviewed in groups of two. The interviews were audio recorded and verbatim transcribed. The interviews were conducted in the Arabic language to be more convenient to the students. Students of the undergraduate program in “name of a university” participated in transcribing the interviews after removing all identifications of the participants. Then, the interviews were translated into English. The participants in the interviews are given pseudonyms to protect their identities. The participants in the interviews have been named: S1, S2, S3, S4, S5, S6 and S7. The questions and answers of the interviews will be discussed in the data analysis section.

**Fig. 1:**
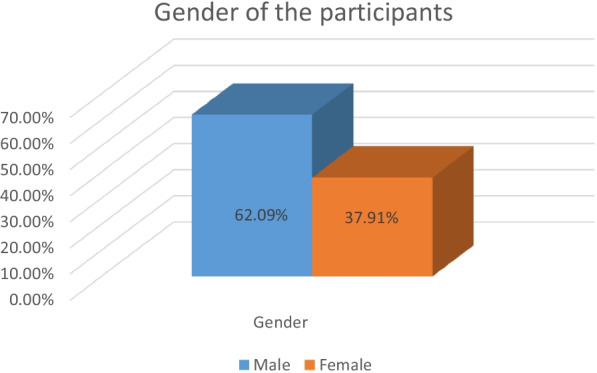
The distribution of the participants according to their gender

With regards to the second phase, online questionnaires were distributed among students of the PYP. Questionnaires were used in this phase as a result of two main reasons. The first reason was the restrictions that were implemented as a response to COVID-19 pandemic. The second reason was making sure that students from both male and female sections of the PYP would be able to participate in this study. As a results, three hundred and ninety-four students from both the male and female sections of the PYP participated in the questionnaire. Figure 1 below shows that that 62.09% of the participants were males whereas 37.91% of the participants were females.

**Fig. 2:**
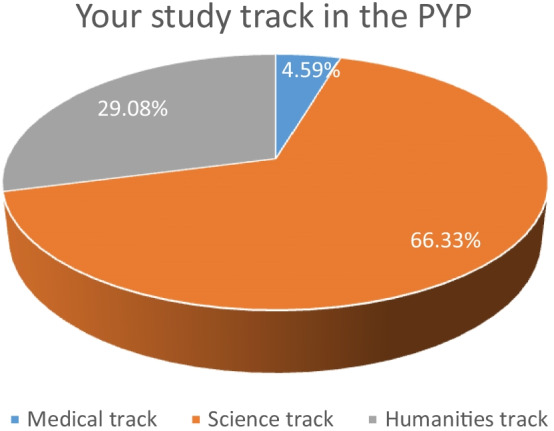
Study track of the participants

The participants in the questionnaire were from all the tracks of the PYP including: the science, the medical, and the humanities tracks to ascertain that all the tracks of the PYP are represented in this study. Figure 2 below indicates that 66.33% of the participants were from the science track, 29.08% of the participants were from the humanities track and the remaining 4.59% of the participants were from the medical track.

The questionnaires were answered by the students according to their convenience and their class schedule. The items of the questionnaire were written in both the Arabic language and the English language to be more convenient to the students. The questionnaire was first piloted to be tested and to be certain that its language is clear. Necessary amendments were made to the questionnaire after piloting it.

All the participants in this study were given the opportunity to withdraw from this study after explaining it to them and asking them to sign a consent form. Data analysis of this study concentrated on analyzing interviews with students of the PYP and questionnaire that were answered by students of the PYP. Interviews were verbatim transcribed using a word processor software with an interest in the words of the participants. Then, the interviews and the questionnaire were analyzed to investigate the participants’ perspective on the LEP of the PYP towards L1. Data analysis included deep reading of all the interview transcripts and the questionnaires. This assisted in generating general comments and observations on the interviews and the questionnaires to gain wider and clearer view of the data that would reflect the diversities within the participant’s perspectives without interference of my interests.

## Data analysis

Data analysis of this study were divided into two phases. The first phase consists of interviews with the participants that were conducted in the period between January and April 2015. The second phase consists of a questionnaire that was answered by the participants in November 2020. Data analysis will begin with the first phase and, then, it will move on to the second phase.

### The first phase

At the beginning, the participants were asked about their understanding of the LEP of the PYP towards using L1 in teaching L2. The extracts below point out the participants’ understanding of this policy.

*Extract*

- **R:**  < What is the policy of the preparatory year program towards using the Arabic language? I mean do they allow the Arabic language? I do not mean the teachers. I mean the administration that is the Dean, the Director of the English language program or the system? I mean are the teachers allowed to use the Arabic language or not? > 
- **S1:**  < The system allows teachers to use the Arabic language! I know it is banned. From what I know the administration bans teachers from using the Arabic language inside the class, except in rare situations of course > .

S1 in this extract reported that, according to his knowledge, the LEP of the PYP bans teachers of L2 from using L1 inside the classroom except in some rare situations. This extract indicates that S1 was not certain about the LEP of the PYP towards L1.

*Extract*

- **S2:** بالنسبة للغة الإنجليزية لا، أما المواد الثانية مسموح… على حسب علمي < In regards to the English language, but it is allowed in the other modules… as far as I know > .
  In this extract, S2 indicated that to the best of his knowledge, the LEP of the PYP bans L1 in the EFL classes whereas it permits it in the modules. This extract points out that S2 was not certain about the LEP of the PYP towards L1.
*Extract*
- **S3:** أتوقع انه مسموح لأنه لمصلحتنا < I expect that it is allowed because it is for our interest > .
  In this extract, S3 anticipated that the LEP of the PYP permits L1 in the EFL classes because this serves the interest of the students of the PYP. This extract shows that S3 was not certain about the LEP of the PYP towards L1.
*Extract*
- **S4:** اللغة العربية ممنوعة منعاً باتاً، حتى في المواد الثانية كل شيء إنجليزي < The Arabic language is strictly banned, even for the other modules, everything is in the English language > .

S4 declared in this extract that L1 is strictly banned by the administration of the PYP in all the modules whether it was the EFL module or the other modules. S4 insisted that L2 only is permitted by the administration of the PYP. This extract highlights that S4 was familiar with the LEP of the PYP’s ban on L1.

*Extract*

- **S5:** في كلاسات اللغة الإنجليزية، اللغة الإنجليزية بس < In the English language classes, the English language only > .
- **R:** ممنوع العربي < The Arabic language is banned > ?
- **S5:** ممنوع العربي، بس في المواد الثانية لا، في بعض الدكاترة يتكلمون عربي عادي < The Arabic language is banned, but for the other modules no, some of the doctors normally speak Arabic > .
- **R:** يعني هو المدرس نفسه < You mean the teacher himself > ?
- **S5:** هو الدكتور نفسه يتكلم عربي < The doctor himself speaks Arabic > .
- **R:** طيب لو شافوه يتكلم عربي؟ مثلاً جاء العميد وشافه يتكلم عربي مثلاً < What if they saw him speaking Arabic? For example, the Dean came and saw him speaking Arabic > ?
- **S5:** أتوقع فيها مشكلة، لأن المادة تدرسها في الإنجليزي < I expect that it will be a trouble because the module is taught in the English language > 

In this extract, S5 reported that L1 is banned in the EFL modules by the administration of the PYP. On the other hand, S5 noted a regular tendency of using L1 by some of the teachers of the other modules. S5 predicted that this use of L1 may cause trouble for the teachers by the administration of the PYP. This extract points out that S5 was aware that the LEP of the PYP bans L1, but he noted that some EFL teachers disregarded this ban.

*Extract*

- **S6:** أتوقع ممنوع < I expect it is banned > 

S6 in this extract revealed that he was not certain about the LEP of the PYP towards using L1. S6 anticipated that L1 is banned by the LEP of the PYP.

*Extract*

- **S7:** والله مايسمحون. هو مايسمحون يعني كا يعني كتحدث يعني بشكل يعني كبير، لكن كمساعدات < they do not allow it. They do not allow it to be spoken in a large amount, but as a kind of help by > [named his EFL teacher] يساعدنا باللغة العربية < he assists us using the Arabic language > 

In this extract, S7 hypothesized that the LEP of the PYP bans L1 unless it was necessary to assist students. S7 gave an example of his EFL teacher’s use of L1 to assist students in understanding L2. This extract indicates that S7 was not certain about the LEP of the PYP towards L1, but he anticipated that the LEP of the PYP permits using L1.

Data analysis of the first phase revealed that six of the participants agreed that the LEP of the PYP bans using L1 in teaching L2 whereas only one of them hypothesized that the LEP permits using L1. However, three of the participants admitted that they were not certain about the LEP of the PYP towards L1 where only one of them asserted that the LEP of the PYP strictly bans using L1 in teaching L2. Data from the first phase highlights that students varied in their understanding of the LEP of the PYP. It is possible that the policy-makers in the PYP did not clarify their policy to the students. One of the participants noted that some of the teachers in the PYP tended to use L1 in their classes despite the LEP’s ban on L1. This student predicted that such teachers would be punished by the administration of the PYP for violating the LEP. As Raschka et al. (2009) have argued, policy-makers tend to treat the use of L1 in teaching L2 as a negative behavior that has to have negative consequences.

The participants were also asked about their opinion regarding the role of L1 in teaching L2 in the LEP of the PYP. The participants’ opinions about this policy are discussed in the extracts below.

*Extract*

- **S1:**  < In regards to this policy, it depends on the type of students that you are teaching. I mean, for example, if you are teaching > Level one  < it is difficult to speak with them in English only, because some of your students in > level one  < do not distinguish between the letters. How would you speak with them in the English language only? But in the higher levels > Level three و < and > two  < and higher than that, you should speak with them in the English language for them to develop. This is my view, that the lower levels because… or, for example, some of the tracks such as the humanities track who really lack the language, you should talk with them in the Arabic language to deliver the information in the correct manner. I think that some of the levels, it is mandatory that you do not ever speak with them in the Arabic language > 

S1 in the above extract stressed that the LEP of the PYP towards L1 should consider the linguistic proficiency level of students in L2. S1 advocated permitting the use of L1 by the LEP of the PYP with students who have low linguistic proficiency whereas he supported banning L1 on advanced level classes. This extract highlights that S1 did not support strict ban of using L1 by the LEP of the PYP.

*Extract*

- **S2:** بالنسبة للمواد الثانية اتفق صح < In regards to the other modules, I agree it is right > .
- **R:** انت تتفق انه المفروض يستخدم العربي < You agree that they should use the Arabic language > ?
- **S2:** صح < that is correct > .
- **R:** والإنجليزي < and the English language module > ?
- **S2:** الإنجليزي! لا، الافضل انهم يتكلمون انجليزي < The English language module! No, it is better for them to speak in the English language > 

In the above extract, S2 supported the LEP of the PYP’s permission of L1 in the modules that use the English language as a medium of instruction. On the other hand, S2 advocated banning L1 in the EFL classes. This extract indicates that S2 supports strict ban on L1 by the LEP of the PYP in the EFL module. On the other hand, S2 supports permitting the use of L1 by the LEP of the PYP in the other modules.

*Extract*

- **S3:**  < I totally agree with him I mean the language… in the English language modules, they should not say any Arabic language words. It is better. whereas for the other modules, physics, chemistry and biology it is difficult to communicate. You have to speak in the Arabic language. I think it is normal to speak in the Arabic language > 

The above extract indicates that S3 agreed with strictly banning L1 by the LEP of the PYP in EFL classes, but he refuted banning L1 in the other modules that use L2 as the main medium of instruction.

*Extract*

- **R:** هل تتفقون مع هذه السياسة، يعني ممنوع العربي داخل الفصل < Do you agree with this policy? I mean banning the Arabic language inside the classroom > ?
- **S4:**  < Look… I mean if we were established correctly before, it is OK. But currently, no. We need, I do not know anything in the English language. I came from high school to study > (XXXX)  < I do not have anything. Therefore, there has to be some compromise. Teach us using some Arabic language and some English language to practice and interact with you and to deliver the correct information. For Some of the students, the class ends and he leaves listening to his doctor only > 
- **R:** طيب ليه يجي يدرس ويطلع < So why he comes to study and leaves > ?
- **S4:** هو جاء يتعلم. لكن المعلومة المهمة. ما تقدر، توصل له المعنى والفكرة أبداً بالإنجليزي < He is coming to learn. But the significant information. You cannot ever deliver the meaning and the idea to him using the English language > 

S4 in the above extract disagreed with the LEP of the PYP towards using L1 in the EFL classes. S4 argued that the LEP of the PYP did not consider the proficiency level of the students in L2. S4 revealed that most students begin the PYP with low proficiency in L2. Therefore, he advocated permitting L1 to assist students in comprehending the classes and interacting with their teachers. S4 in this extract supports permitting L1 by the LEP of the PYP.

*Extract*

- **S5:** زين انهم يتكلمون ما يتكلمون عربي < It is good that they do not speak the Arabic language > .
- **R:** يعني انتم حتى ب < You mean even in > level one ترى المفروض ما يتكلمون عربي < you think that they should not speak the Arabic language > ?
- **S5:** لا، ما يتكلمون عربي < No, they should not speak the Arabic language >. Level one  < It is better for doctors to use simple words and a lot of signals for us to understand correctly. In the first and second weeks, we will not understand, but after that we will > (XXXX)

In the above extract, S5 supports the LEP of the PYP’s ban on L1. S5 recommended using various tools such as simple language and body gestures to assist students in comprehending the lessons instead of using L1. S5 felt that this would assist in providing students with gradual improvement of their proficiency in L2.

*Extract*

- **R:** هل تتفق مع هذه السياسة؟ هل تتفق والله المفروض انه ممنوع اللغة العربية بالفصل < Do you agree with this policy? Do you agree with that the Arabic language is supposed to be banned inside the classroom > ?
- **S6:** لا والله ما اتفق. لأنه به بعض الكلمات المفروض أنه تترجم علشان يفهم الطالب وش معنى الكلمة < I do not agree. Because some words are supposed to be translated for the student to understand the meaning of these words > 

The above extract indicates that S6 did not agree with the LEP of the PYP towards L1. S6 hypothesized that using L1 in teaching L2 would assist students in comprehending L2.

*Extract*

- **R:** هل تتفق معها بأن الادارة ما تسمح باللغة العربية < Do you agree with the administration in banning the Arabic language > ?
- **S7:**  < I believe it is the best, it is better in the languages, I mean for example in the classes of the English language, biology, and physics, it is better I mean not to speak in the Arabic language > 

S7 in the above extract supports the LEP of the PYP towards L1. In addition, S7 advocates banning L1 in all the modules including the EFL module.

In the first phase of this study, the participants revealed their opinions regarding the ban of the LEP of the PYP on L1. Four of the participants supported the LEP of the PYP towards L1. These four participants contended that the LEP of the PYP should ban L1 in teaching L2. Simon (2001) noted that students usually use L1 with a sense of guilt. One of the participants suggested a few alternative methods to be used instead of using L1. Ellis (1984) suggests that using L1 would deprive students from significant input in L2. Two of these four participants called for the LEP of the PYP to permit using L1 in all the modules of PYP except the EFL module whereas one of the participants advocated a strict ban on L1 by the LEP in all the modules of the PYP without any exception. On the other hand, three of the participants disagreed with the LEP of the PYP towards L1. These three participants suggested that the LEP of the PYP should permit using L1 in teaching L2. Two of these students urged the LEP of the PYP to consider the proficiency level of the students of the PYP. These two students advocated permitting L1 in the beginner classes. Tien (2009) contends that L2-only policy is not feasible with students who have low proficiency in L2. Two of the students asserted that permitting L1 in teaching L2 would assist students in improving their comprehension. The use of L1 assists in improving students’ comprehension (see for example Edstrom, 2006; Kim & Elder, 2005; Liu et al., 2004; Uys & Dulm, 2011). Students are variant in presenting their opinions regarding the LEP of the PYP, and this may differ according to their personal experiences.

### The second phase

In the second phase, the participants answered a questionnaire about their understanding of the LEP of the PYP towards using L1 in teaching L2. The graphs below point out the participants’ understanding of this policy.

**Fig. 3:**
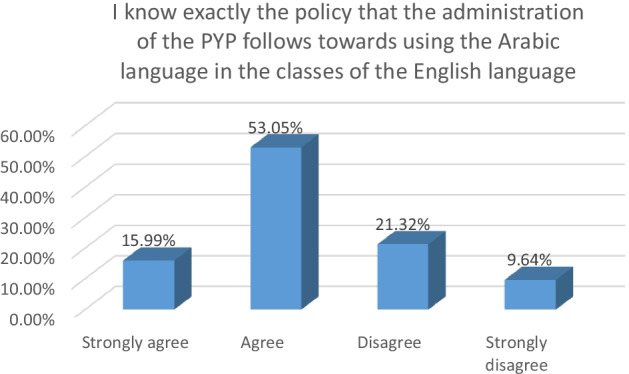
Students’ Knowledge about the LEP of the PYP towards L1

Figure 3 above highlights that 69.04% of the participants claimed that they knew exactly the LEP of the PYP towards using L1 in L2 classes. On the other hand, 30.96% of the participants admitted that they were not certain what the LEP of the PYP towards using L1 in L2 classes was.

**Fig. 4:**
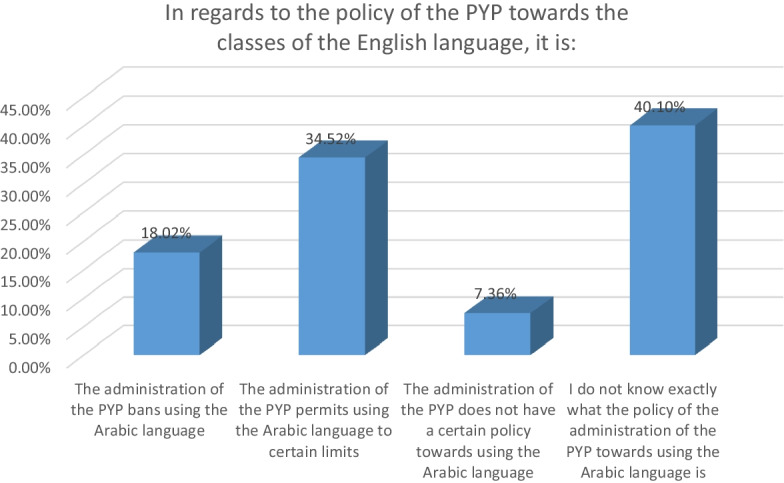
Students’ understanding of the LEP of the PYP towards L1

Figure 4 above reveals that 18.02% of the participants contended that the LEP of the PYP bans using L1 in teaching L2 while 34.52% of the participants claimed that the LEP of the PYP permits using L1 in teaching L2. On the other hand, 7.36% of the participants hypothesized that the PYP did not have a certain policy regarding the use of L1 in teaching L2. 40.10% of the participants conceded that they were not certain about the LEP of the PYP towards using L1 in teaching L2.

The vast majority of the participants in the second phase of this study contended that they knew exactly the LEP of the PYP towards using L1 in teaching L2. However, when the participants were asked what was the LEP of the PYP towards using L1 in teaching L2, they did not agree on the nature of the LEP of the PYP towards L1. Spolsky (2004) illustrates that the impact of language policy on language practice cannot be guaranteed or be consistent. Most of the participants conceded that they were not certain about the LEP of the PYP towards L1. A significant number of the participants anticipated that the LEP of the PYP permits using L1 in teaching L2. A relatively small number of the participants agreed that the LEP of the PYP bans using L1 in teaching L2. Data from the second phase of the study point out that most of the students did not know the LEP of the PYP towards L1. Bassiouney (2009) point out that it is not feasible for a language policy to be implemented if it is not in line with language practice. It is possible that the policy-makers did not clarify the policy to their students.

**Fig. 5:**
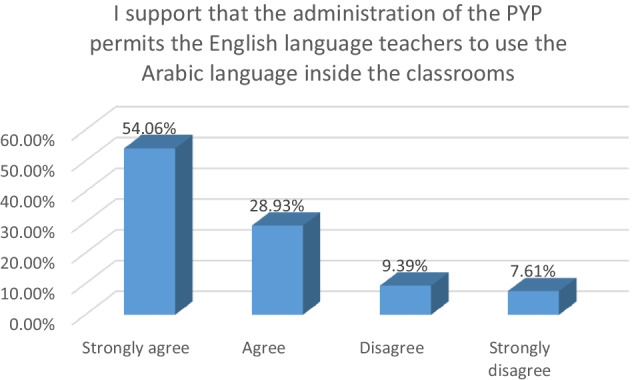
Students’ attitude towards the LEP of the PYP towards L1

In Fig. 5 above, 82.99% of the participants supported the suggestion for the LEP of the PYP to permit teachers of L2 to use L1 in teaching L2. On the other hand, only 17% of the participants disagreed with this suggestion.

The vast majority of the participants in the second phase of this study agreed with the suggestion that the LEP of the PYP should permit teachers to use L1 in teaching L2. Alqahtani (2022) revealed that the majority of Saudi students support using translanguaging in the EFL classes.

## Discussion

Data from the first and second phases of this study point out that the majority of the students were not certain about the LEP of the PYP towards L1. Not including the perspective of students in LEP of the PYP is in line with Hornberger and Johnson’s (2007) viewpoint that language policies are useless if humans do not play a crucial role in implementing them. Despite the long period between collecting data for the first and second phases, it could be anticipated from the results of this study that the policy-makers in the PYP did not take any effective actions that would assist in enhancing students’ awareness about the LEP of the PYP towards using L1 in teaching L2. This indicates a significant discrepancy between the imagined policy and its implementation as noted by Lo Bianco and Aliani (2013). This is also consistent with Alomaim (2019) finding that the policy-makers were treating this LEP as a de facto policy that it did not need to be written or informed to teachers or, as the findings of this study indicates, to students as well. However, the literature shows that language policies occur whether they have been clarified by the authority or not (see for example Bassiouney, 2009; Spolsky, 2004).

Data from the first phase of this study indicate that most of the students did not support permitting the use of L1 by the LEP of the PYP to teach L2 whereas data from the second phase of this study point out that most of the students supported permission of L1 by the LEP of the PYP to teach L2. According to research by Lo Bianco and Aliani (2013), students actively form opinions about the implementation of policies. The literature highlights that language policy cannot be guaranteed to be reflected by language practice (see for example Bassiouney, 2009; Spolsky, 2004). Data of this study reveals that the general perspective of students of the PYP towards the role of L1 in teaching L2 has changed over the years. This may be influenced by the pandemic of COVID-19 and the switch from attending classes in person to distance learning. This switch led to loosing face-to-face interaction between students and their teachers. Therefore, students may felt that they needed different tools to interact with their teachers that include L1. This support for using L1 agrees with Garton’s (2014) argument that permitting L1 may improve students’ opportunities of learning. Creese (2005 cited in Asker & Martin-Jones, 2013, p. 345) points out that teachers and students use several creative strategies to deal with the limitations of language policy.

## Conclusion

This section will answer the questions of this study. In terms of the first question of this study, data analysis shows that most of the students of the PYP were not certain about the LEP of the PYP towards L1. Although there was a period of around four years in collecting data for the two phases of this study, the policy-makers in the PYP still did not pay particular attention to clarifying the LEP of the PYP towards L1.

In regards to the second question of this study, data analysis shows that students’ opinion towards using L1 in teaching L2 in the first and second stages of this study did not correlate. Data analysis indicates that most of the students in the first phase of the study did not support using L1 in teaching L2 whereas the majority of students in the second phase of this study leaned towards supporting the use of L1 in teaching L2. It is possible that COVID-19 pandemic and the educational sector’s shift towards distance learning have affected students’ opinion towards the significance of using L1 in teaching L2. Students lacked face-to-face interaction with their teachers as a result of shifting to distance learning. Therefore, it is possible that students felt that they needed every available tool, including L1, that would assist them in learning L2.

This study’s main contribution to knowledge is investigating students’ perspective towards the LEP of the PYP towards using L1 in teaching L2. Data analysis of this study points out that policy-makers in the PYP have not considered students’ perspective in the LEP of the PYP towards using L1 in teaching L2. This study urges policy-makers in the PYP to include students’ perspective in the LEP of the PYP. This could be accomplished through conducting workshops that would include students to include their voice in the LEP of the PYP towards using L1 in teaching L2. The LEP of the PYP should consider students as bilingual speakers of the two languages (see for example Liebscher and Dailey-O’Cain, 2005). Therefore, the LEP of the PYP should adopt an “English-mainly” policy instead of its English-only policy (see for example McMillan & Rivers, 2011). However, the LEP of the PYP should not lead to overuse of L1 (see for example Turnbull & Dailey-O’Cain, 2009). This study was conducted in the PYP where university students are prepared for their undergraduate education. Further study should be conducted in the undergraduate programs to investigate the LEP of the undergraduate courses towards using L1 in teaching courses that mainly use L2 as their main medium of instruction.

## Data Availability

The data in this work is available for reviewers on request at any time.

## Abbreviations

L1: First language
L2: Second language
LEP: Language education policy
PYP: Preparatory year program
EFL: English as a foreign language
